# EdgeLane-SEG: an energy-efficient embedded edge AI framework for real-time road marking and lane lines detection with instance segmentation in ADAS and autonomous driving

**DOI:** 10.3389/frai.2026.1872217

**Published:** 2026-08-05

**Authors:** Mohammed Chaman, Anas El Maliki, Wiame Bouyoussef, Abdelmounaim Belaaribi, Younes Laababid, Zouhair Sadoune, Abdelkader Mezouari, Hamad Dahou, Abdelkader Hadjoudja

**Affiliations:** 1 Laboratory of Electronic Systems Information Processing Mechanics and Energetics, Ibn Tofail University, Kenitra, Morocco; 2 Laboratory of Scientific Research and Innovation, Ibn Tofail University, Kenitra, Morocco; 3 Laboratory of Computer Science and Interdisciplinary Physics Normal Superior School Fez, Sidi Mohamed Ben Abdellah University, Fes, Morocco

**Keywords:** ADAS, detection and instance segmentation, edge computing, embedded AI, energy efficiency, road markings and lane lines

## Abstract

**Objective:**

Accurate and energy-efficient perception of road-surface markings is essential for Advanced Driver Assistance Systems (ADAS) and autonomous driving, particularly under real-time embedded constraints. This study proposes EdgeLane-SEG, a unified framework designed to achieve high instance segmentation accuracy while maintaining low computational cost and power consumption on resource-constrained edge platforms.

**Methods:**

The proposed framework integrates two single-stage models, YOLO11-SEG and YOLO26-SEG, for joint object detection and instance segmentation. A dedicated dataset of 10,542 annotated images with 23,420 labeled instances was constructed, covering lane markings, directional arrows, and pedestrian crossings. Both models were trained under identical conditions using a unified multi-task loss combining IoU-based regression, objectness, classification, and hybrid Binary Cross-Entropy and Dice segmentation losses. Performance was evaluated using precision, recall, F1-score, mAP@0.5, mAP@0.5–0.95, FPS, and FPS/W. Deployment was conducted on NVIDIA Jetson Nano, Raspberry Pi 5, Raspberry Pi 5 with Intel Movidius VPU, and Raspberry Pi 5 with Hailo-8 NPU.

**Results:**

Both models achieved high detection and segmentation performance, with mAP@0.5 exceeding 98%. YOLO26-SEG demonstrated superior inference speed and energy efficiency across all platforms, achieving higher FPS and FPS/W than YOLO11-SEG. The Hailo-8 NPU configuration achieved the best embedded performance, reaching 50–52.6 FPS and 18.85 FPS/W for YOLO26-SEG.

**Conclusion:**

EdgeLane-SEG effectively balances accuracy, efficiency, and deployment feasibility. YOLO26-SEG with Hailo-8 NPU acceleration is particularly suitable for real-time embedded ADAS applications, enabling energy-efficient and reliable road perception in resource-constrained environments.

## 1 Introduction

Advanced Driver Assistance Systems (ADAS) and autonomous driving technologies have become central components of intelligent transportation systems, driven by the need to improve road safety, reduce driver workload, and enable reliable vehicle autonomy. A fundamental requirement of these systems is robust real-time perception, which allows vehicles to interpret complex road environments and support safety-critical functions such as lane keeping, lane departure warning, trajectory planning, localization, and decision-making. Among road perception tasks, the accurate detection and segmentation of lane lines and road-surface markings is particularly important, since these visual cues define drivable regions, guide vehicle positioning, and provide semantic information for navigation in structured and semi-structured traffic environments (Medina et al., 2026; Hashimoto et al., 2026; Bruno et al., 2023).

Early lane and road marking perception systems mainly relied on classical image processing techniques, including edge detection, Hough transform, color thresholding, handcrafted feature extraction, inverse perspective mapping, and geometric line fitting. These approaches are computationally efficient and remain attractive for low-cost embedded systems; however, they are often sensitive to illumination changes, shadows, worn markings, occlusions, curved roads, and adverse weather conditions. Recent work on deterministic CPU-only lane detection has shown that geometry-driven methods can still be useful for safety-critical and cost-constrained ADAS applications, especially when dedicated accelerators are unavailable, but their robustness remains limited in highly variable real-world scenes (Tsai et al., 2026). These limitations have motivated the adoption of deep learning-based perception models capable of learning discriminative visual features directly from data.

Deep learning, particularly convolutional neural networks (CNNs), has significantly improved object detection, semantic segmentation, and instance segmentation performance in road scene understanding (Sayyad and Attarde, 2026; Biswas and Wang, 2023). Semantic segmentation-based lane detection networks have demonstrated strong performance for clear and occluded roads, nighttime driving, and curvy road scenarios. For example, InSegNet introduced encoder–decoder variants with inception and residual connections to enhance lane feature extraction and improve robustness under clear, occluded, day, and night conditions (Shaikh and Faraz, 2026). Similarly, CNN-based lane detection combined with YOLO-based object recognition and Grad-CAM visualization has been proposed to improve real-time scene understanding and model interpretability for autonomous driving applications (Gupta et al., 2026). Although these studies demonstrate the value of learning-based lane perception, many approaches focus mainly on semantic lane segmentation or object detection, while fewer works jointly address instance-level segmentation of multiple road-surface primitives, including lane boundaries, directional arrows, and pedestrian crossings.

Instance segmentation is particularly relevant for ADAS because it provides both object-level localization and pixel-level mask delineation. Unlike semantic segmentation, which assigns pixels to general classes, instance segmentation can distinguish separate lane markings or adjacent road-surface elements. This capability is important in multi-lane roads, intersections, and dense urban environments where near/far lane boundaries, arrows, and pedestrian crossings may overlap visually or appear with similar geometric patterns. However, accurate instance segmentation of road markings remains challenging because these structures are often thin, elongated, partially occluded, low-contrast, and highly affected by perspective distortion.

In parallel, the YOLO family of models has become widely adopted for real-time perception because of its favorable balance between accuracy and inference speed. Recent YOLO-based architectures have progressively improved detection and segmentation through anchor-free prediction, redesigned detection heads, multi-scale feature fusion, attention mechanisms, and more efficient deployment-oriented designs (Caballero-Ramírez et al., 2025; Santos dos Santos Júnior et al., 2025; He et al., 2025; Khanam and Hussain, 2024; Tian et al., 2025). In this context, YOLO11-SEG provides a strong single-stage detection–segmentation baseline suitable for real-time perception, while YOLO26-SEG introduces more deployment-oriented improvements, including streamlined inference, NMS-free prediction, and optimization mechanisms intended to reduce latency and improve energy efficiency on embedded platforms (Hidayatullah and Tubagus, 2026).

Despite these advances, deploying deep learning-based instance segmentation models in real-time embedded ADAS systems remains a major challenge. Embedded platforms operate under strict constraints related to latency, memory usage, computational resources, thermal stability, and power consumption. High-performance GPUs used during model training are generally unsuitable for low-cost in-vehicle deployment. Therefore, practical ADAS perception requires not only high detection and segmentation accuracy, but also hardware-aware optimization and systematic benchmarking across heterogeneous edge platforms, including CPU-, GPU-, VPU-, and NPU-based configurations.

To address these challenges, this paper proposes EdgeLane-SEG, an energy-efficient embedded edge AI framework for real-time road marking and lane line detection with instance segmentation. The framework adopts a dual-model evaluation strategy based on YOLO11-SEG and YOLO26-SEG. In this work, the term dual-model does not refer to a fused or ensemble model; rather, it indicates that two segmentation architectures are trained, optimized, deployed, and evaluated independently under identical experimental conditions to ensure a fair and reproducible comparison. This design enables a clear assessment of the trade-offs between segmentation accuracy, inference speed, energy efficiency, and hardware scalability.

The proposed framework is evaluated using a dedicated road-surface dataset containing lane markings, directional arrows, and pedestrian crossings. Unlike approaches that focus only on lane boundaries, EdgeLane-SEG targets multiple road-surface primitives required for practical ADAS perception. In addition, the trained models are deployed on several embedded platforms, including NVIDIA Jetson Nano, Raspberry Pi 5, Raspberry Pi 5 with Intel Movidius VPU, and Raspberry Pi 5 with Hailo-8 NPU. This comprehensive embedded deployment refers to a complete hardware-aware evaluation pipeline involving model conversion, platform-specific optimization, runtime benchmarking, and energy-efficiency analysis using metrics such as latency, FPS, and FPS/W.

The main contributions of this work are summarized as follows:

- A dual-model evaluation framework integrating YOLO11-SEG and YOLO26-SEG for real-time road marking and lane line detection with instance segmentation.
- A dedicated instance-level road-surface dataset covering lane boundaries, directional arrows, and pedestrian crossings under diverse driving conditions.
- A hardware-aware embedded deployment strategy across CPU-, GPU-, VPU-, and NPU-based platforms, including Jetson Nano, Raspberry Pi 5, Intel Movidius VPU, and Hailo-8 NPU.
- A systematic comparison of accuracy, segmentation quality, inference speed, latency, and energy efficiency to identify suitable model–hardware configurations for real-time ADAS deployment.

By combining instance segmentation accuracy with embedded edge optimization, this study bridges the gap between high-performance deep learning perception and practical in-vehicle deployment constraints. The remainder of the paper presents the related work, proposed methodology, dataset preparation, model architecture, embedded deployment strategy, experimental results, and discussion.

## Related work

Lane line and road marking perception remains a core requirement for Advanced Driver Assistance Systems (ADAS) and autonomous driving because it directly supports lane keeping, trajectory planning, and scene understanding. Earlier solutions were dominated by classical vision pipelines that apply handcrafted preprocessing (e.g., smoothing, thresholding, edge detection), followed by geometric line fitting and Hough-based extraction. For instance, Javeed et al. proposed an Otsu-thresholding and Canny/Sobel-based fast Hough transform approach that improves reasoning speed and detection stability in complex road conditions, emphasizing the role of region-of-interest selection and least-squares fitting for lane tracking (Javeed et al., 2023). While such pipelines can be efficient, they are inherently sensitive to shadows, worn markings, illumination changes, and occlusions, which motivates deep learning alternatives that learn robust features from data.

Within deep learning, lane-related perception has evolved along two principal directions: semantic segmentation and instance-level segmentation. Semantic segmentation methods typically predict pixel-wise classes (lane/drivable area vs. background) and are attractive for their conceptual simplicity and end-to-end optimization. Wang et al. introduced a semantic segmentation framework with a backbone–neck–head design using feature pyramid networks (FPN) and dedicated heads for drivable area and lane line segmentation, validated on BDD100K, illustrating the effectiveness of multi-scale feature fusion for lane-related tasks (Wang et al., 2024). However, semantic outputs can struggle when multiple lane instances need to be separated (e.g., adjacent lane lines, complex multi-lane scenes), where instance segmentation becomes more appropriate.

Instance segmentation for lane lines has therefore gained substantial attention, especially for dense or ambiguous scenarios where multiple lane markings must be distinguished reliably. Cheng et al. proposed InstLane, described as a benchmark for lane instance segmentation with laterally mounted cameras, and introduced GeoLaneNet, a geometry-aware network designed to reduce missing or duplicate detections in dense-lane scenarios and accelerate inference via architectural choices; their experiments highlight the importance of structural priors and larger receptive fields for lane topology learning (Cheng et al., 2024). In a similar vein, Cheng et al. presented an instance-segmentation-based lane line detection algorithm targeting challenging scenes with blurred roads and sparse/slender lane structures; they improved feature extraction using a lightweight RepVGG-A0 encoder, proposed asymmetric shuffling convolutions, and employed adaptive upsampling, then demonstrated embedded deployment on Jetson Nano with TensorRT half-precision acceleration—reporting strong accuracy and real-time performance after deployment optimization (Cheng et al., 2023). These works collectively show that lane perception benefits from instance-level reasoning and that embedded feasibility depends on careful architecture design and deployment-level acceleration.

Beyond lane lines, road scene understanding often requires detecting and segmenting multiple road-surface elements, including arrows and pedestrian crossings, as well as traffic symbols. Although road-surface traffic sign segmentation is less explored than upright traffic sign detection, Zhang et al. proposed an instance segmentation model (RTS R-CNN) derived from Mask R-CNN to handle small pixel-ratio targets and limited datasets, improving macro F1-score while maintaining practical inference speed through feature extraction and fusion enhancements plus augmentation strategies (Zhang et al., 2023). Such studies reinforce the observation that segmentation tasks on small or thin targets are sensitive to feature fusion quality and multi-scale design—considerations that also apply to lane markings and directional arrows.

In parallel, YOLO-based architectures have become prominent due to their favorable trade-off between accuracy and speed, which is crucial for real-time ADAS. Recent research has explored YOLO variants for segmentation, especially lightweight designs optimized for edge platforms. PSC-YOLO was introduced as a lightweight instance segmentation approach for urban roads, emphasizing multi-scale feature learning and efficient mask decoding inspired by SAM-like principles to balance segmentation quality and real-time throughput, outperforming YOLOv8n-seg in mask AP while achieving high FPS (Gu and Zhang, 2025). Qu et al. enhanced YOLOv5 for lane line segmentation by integrating multi-scale feature extraction via diversified branch blocks and proposing a segmentation head inspired by YOLACT, with additional tracking components such as Kalman filtering, highlighting a practical path to improve robustness for lane departure warning systems (Feilong et al., 2025). More recently, Liao et al. proposed dual-path enhancements to YOLO11 for lightweight instance segmentation, combining attention mechanisms (e.g., SimAM) and efficient convolutions (SPD-Conv), along with improved upsampling, demonstrating measurable gains in mAP and reduced complexity on Cityscapes—an important result for resource-constrained deployment (Liao et al., 2025). At a system level, Teboulbi et al. proposed a dual-YOLOv8 fusion strategy that combines complementary segmentation models via multi-scale fusion to improve instance-aware semantic segmentation, and they further discussed embedded feasibility on edge ARM/GPU platforms, supporting the broader idea that multi-model strategies can strengthen robustness when resources permit (Teboulbi et al., 2026).

Embedded deployment is increasingly treated as a first-class research objective rather than a post-processing step. Several studies explicitly quantify the accuracy–latency–resource trade-offs on embedded hardware. Ramos-Sanchez et al. retrained SSD-based detectors directly on an NVIDIA Jetson platform and discussed dataset-driven trade-offs between accuracy and computational cost for embedded ADAS in congested urban environments, showing that model choice and input resolution materially affect feasibility under deployment constraints (Ramos-Sanchez et al., 2026). DriveRight introduced a Raspberry Pi-based embedded hazard detection and alert system, comparing detectors such as Faster R-CNN and MobileNet-SSD and validating field performance under varying conditions, thereby illustrating end-to-end feasibility using low-cost embedded devices while emphasizing real-time and power constraints (Alsayaydeh et al., 2026). Related deployment-oriented work on traffic sign detection also highlights the role of optimized one-stage detectors and embedded acceleration; Nandy and Dharmesh describe embedded implementation considerations and discuss tuning for real-time inference on NVIDIA Jetson Xavier NX, reinforcing the practicality of embedded deep models when the pipeline is engineered end-to-end (Nandy and Dharmesh Kirit, 2026). Farhani et al. further show that combining attention and Transformer modules within a YOLO-based architecture can improve robustness in complex visual environments, while still enabling embedded real-time deployment via TensorRT optimization demonstrating that accuracy-oriented architectural improvements can remain deployable with appropriate optimization tooling (Farhani et al., 2026). For road anomaly perception on low-cost devices, Martins et al. evaluated multiple detectors and reported that YOLOv11 provides a strong accuracy–efficiency compromise, including feasibility on Raspberry Pi, which aligns with the broader trend of using modern YOLO variants to satisfy real-time constraints on constrained hardware (Martins et al., 2025). Transformer-based detection is also moving toward edge feasibility; Aljahani’s RT-DETR edge deployment study for distracted driving shows that transformer detectors can be adapted with deployment-oriented optimizations to sustain real-time performance on ARM-based devices, suggesting a broader convergence toward efficient edge inference across model families (Aljahani, 2025).

System integration studies further emphasize that perception models must connect to decision-making and planning pipelines. Nguyen et al. presented a multi-sensor fusion and hierarchical decision-making architecture for autonomous vehicles under mixed traffic conditions, integrating segmentation and LiDAR cues to support both long-term planning and short-term reactive planning, which illustrates the importance of perception outputs that remain stable across scenarios and can be consumed by downstream modules (Nguyen et al., 2025). Within this broader ADAS context, our recent research has focused on embedded feasibility and reproducible benchmarking. We previously introduced a real-time vehicle detection system based on YOLOv11 deployed on Jetson Nano and Raspberry Pi 5, demonstrating high accuracy with balanced resource consumption under embedded constraints (Chaman et al., 2025a). We also proposed a real-time speed-limit sign detection framework using YOLOv12 optimized via TensorRT and ONNX Runtime for Jetson Nano and Raspberry Pi 5, highlighting the benefit of deployment-aware optimization and robustness-oriented dataset preparation (Chaman et al., 2026b). In addition, our benchmarking study compared recent YOLO versions (YOLOv8–YOLOv12) under unified conditions and evaluated both accuracy and runtime measures, supporting evidence-based selection of architectures for embedded ADAS subsystems (Chaman et al., 2026a). Most directly related to the present work, we presented YOLOv11-SEG for detection and instance segmentation of road markings and lane lines, introducing architectural modules to enhance feature extraction and attention for small or occluded markings, and demonstrating a balanced accuracy–efficiency profile suitable for embedded use (Chaman et al., 2025a). Finally, the emergence of YOLO26 introduces architectural changes targeting faster inference and CPU-friendly execution, including end-to-end NMS-free inference and training/optimization updates aimed at edge devices, making YOLO26-SEG a compelling candidate for next-generation embedded perception pipelines that require both speed and segmentation capability (Hidayatullah and Tubagus, 2026).

Taken together, the literature indicates clear progress in three main directions: moving from classical lane extraction toward deep segmentation for improved robustness, adopting instance segmentation to handle multi-lane and thin-structure delineation, and treating embedded deployment as an integral part of ADAS research. Hardware-aware optimization has become increasingly important, with TensorRT, ONNX Runtime, OpenVINO, VPU acceleration, and Hailo NPU-based inference enabling more practical real-time deployment on resource-constrained platforms. However, there remains a practical need for unified, deployment-oriented studies that jointly evaluate modern segmentation-capable YOLO variants on representative embedded edge platforms while targeting road-surface primitives beyond lane boundaries alone, including directional arrows and pedestrian crossings. This motivates the proposed EdgeLane-SEG framework, which integrates YOLO11-SEG and YOLO26-SEG within a comparative performance analysis and multi-hardware deployment setting, including CPU-, GPU-, VPU-, and NPU-based configurations, thereby aligning methodological rigor with real-world embedded feasibility requirements.

## 2 Methodology

This section describes the methodological framework adopted to develop and evaluate the proposed EdgeLane-SEG system for real-time road marking and lane line detection with instance segmentation in embedded ADAS environments. The research methodology follows a structured pipeline integrating dataset preparation, deep learning model training, multi-task optimization, model conversion, and hardware-aware deployment across heterogeneous edge platforms.

The process begins with the construction of a dedicated dataset containing annotated lane boundaries, directional arrows, and pedestrian crossings. Instance-level annotations are generated to support both object detection and pixel-wise segmentation. The dataset is divided into training, validation, and testing subsets to ensure reliable generalization and unbiased evaluation.

Two single-stage deep learning architectures, YOLO11-SEG and YOLO26-SEG, are trained under identical experimental conditions to enable a fair comparison. A unified multi-task loss function jointly optimizes bounding box regression, objectness confidence, classification, and segmentation mask prediction. Hyperparameters, including input resolution, batch size, learning rate, and optimization strategy, are carefully tuned to ensure stable convergence and robust performance.

Following training, the models are exported and optimized for embedded deployment using TensorRT, ONNX Runtime, OpenVINO, and HailoRT. TensorRT is used for NVIDIA Jetson Nano acceleration, ONNX Runtime for Raspberry Pi 5 CPU-based inference, OpenVINO for Intel Movidius VPU deployment, and HailoRT with Hailo Executable Format (HEF) for Raspberry Pi 5 with Hailo NPU acceleration. Performance evaluation considers both accuracy metrics, including Precision, Recall, and mAP, and runtime metrics, including latency, FPS, resource utilization, and FPS/W. This comprehensive methodology ensures reproducibility and practical feasibility for real-world embedded ADAS applications.

### 2.1 Proposed EdgeLane-SEG approach

**Figure 1:**
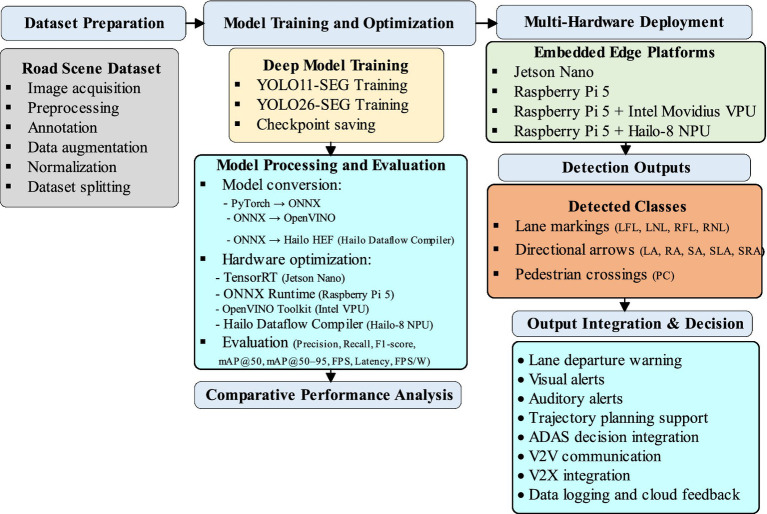
Overview of the proposed EdgeLane-SEG framework.

The proposed EdgeLane-SEG framework is designed to enable real-time detection and instance segmentation of road markings and lane lines for Advanced Driver Assistance Systems (ADAS) and autonomous driving applications. The overall architecture of the proposed methodology is illustrated in Figure 1, which summarizes the complete processing pipeline from dataset preparation to model training, multi-hardware deployment, output integration, and comparative evaluation.

The workflow begins with the dataset preparation stage, including image acquisition from real road scenes, preprocessing, manual annotation, data augmentation, normalization, and dataset splitting. This step ensures robustness against illumination variations, occlusions, and geometric distortions commonly encountered in real-world driving environments.

The second stage consists of model training and optimization, where both YOLO11-SEG and YOLO26-SEG are trained independently under identical experimental conditions. It is important to clarify that the proposed dual-model framework does not refer to a fused model or an ensemble inference architecture. Instead, the term dual-model describes a comparative evaluation strategy in which YOLO11-SEG and YOLO26-SEG are trained, optimized, deployed, and evaluated independently. Therefore, performance measurements are reported separately for each model rather than as a single combined dual-model result. This design enables a fair comparison of the two architectures in terms of detection accuracy, instance segmentation quality, inference speed, latency, and energy efficiency, while preserving the real-time and low-power requirements of embedded ADAS deployment.

Checkpoint saving and validation monitoring are applied to ensure stable convergence, reproducibility, and fair performance comparison. Following training, a dedicated model processing and evaluation module is applied. The trained models are exported from PyTorch to ONNX format to enable cross-platform compatibility. Hardware-specific optimizations are then performed using TensorRT for Jetson Nano, ONNX Runtime for Raspberry Pi 5, OpenVINO Toolkit for Intel Movidius VPU acceleration, and Hailo Dataflow Compiler with Hailo Executable Format (HEF) for Hailo-8 NPU deployment. Performance is evaluated using precision, recall, F1-score, mAP@0.5, mAP@0.5–0.95, FPS, latency, and FPS/W metrics.

The deployment stage targets multiple embedded edge platforms, including Jetson Nano, Raspberry Pi 5, Raspberry Pi 5 with Intel Movidius VPU, and Raspberry Pi 5 with Hailo-8 NPU. This heterogeneous deployment strategy enables systematic benchmarking under real-time, resource-constrained, and power-limited conditions.

The final stage generates structured detection outputs, including lane markings (LFL, LNL, RFL, RNL), directional arrows (LA, RA, SA, SLA, SRA), and pedestrian crossings (PC). These outputs are integrated into downstream ADAS functions such as lane departure warning, visual and auditory alerts, trajectory planning support, ADAS decision integration, V2V communication, V2X integration, and data logging with cloud feedback.

Overall, the proposed EdgeLane-SEG approach combines advanced instance segmentation models with embedded edge acceleration techniques, achieving a balanced trade-off between segmentation precision, inference speed, and energy efficiency. This integration makes the framework suitable for scalable deployment in intelligent transportation systems and real-world autonomous driving scenarios.

### 2.2 Architecture of YOLO11-SEG and YOLO26-SEG

The proposed EdgeLane-SEG framework integrates two single-stage detection and instance segmentation architectures, namely YOLO11-SEG and YOLO26-SEG. Both models are structured according to the Backbone–Neck–Head paradigm and are designed to predict bounding boxes, object confidence scores, class probabilities, and pixel-level instance masks within a unified inference pipeline. Although the two models share the same multi-task learning objective, they differ in architectural refinements, post-processing strategies, and deployment-oriented optimizations that influence computational efficiency and embedded suitability.

**Figure 2:**
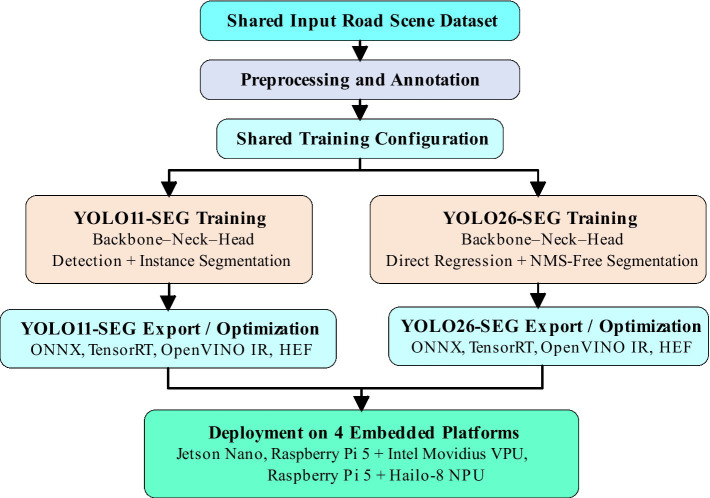
Dual-model training, export, and multi-platform deployment pipeline of the proposed EdgeLane-SEG framework.

To further clarify the dual-model strategy adopted in the proposed EdgeLane-SEG framework, Figure 2 illustrates the complete training, export, optimization, and multi-platform deployment pipeline. The term dual-model does not refer to a fused or ensemble model. Instead, YOLO11-SEG and YOLO26-SEG are trained, optimized, exported, and deployed independently under identical experimental conditions, allowing a fair comparison in terms of detection accuracy, instance segmentation quality, inference speed, latency, power consumption, and energy efficiency.

**Figure 3:**
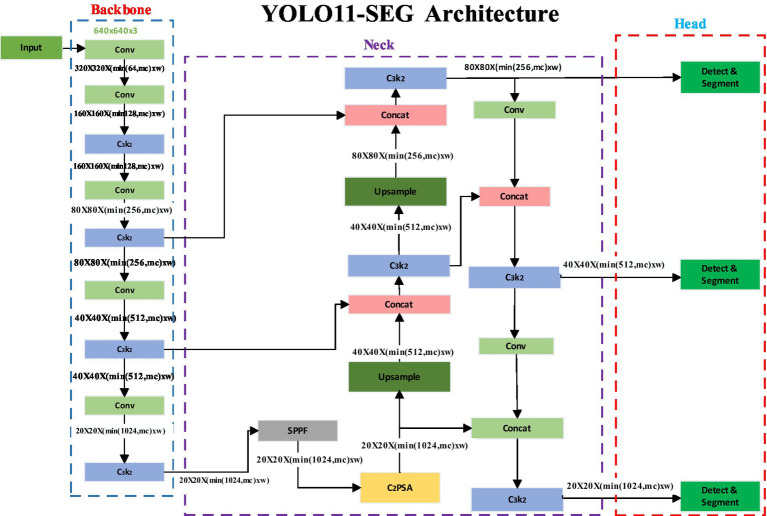
Architecture of YOLO11-SEG network.

YOLO11-SEG follows a conventional hierarchical feature extraction strategy, as illustrated in Figure 3. The backbone processes the 640 × 640 input image through successive convolutional layers and C2f modules, progressively reducing spatial resolution while increasing semantic depth. A Spatial Pyramid Pooling Fast (SPPF) block is incorporated to enlarge the receptive field and enhance contextual representation, which is particularly important for elongated and thin road structures such as lane boundaries and directional arrows. The neck employs a feature pyramid-based design with multi-scale feature fusion through upsampling and concatenation operations. This structure strengthens robustness against scale variation, partial occlusion, and perspective distortion in complex driving environments. The detection–segmentation head operates in an end-to-end manner, producing bounding box predictions and instance masks simultaneously. Detection and segmentation branches share backbone and neck features, ensuring efficient computation while preserving high segmentation fidelity (Chaman et al., 2025b; Ultralytics, 2025a, 2025b; Sapkota and Karkee, 2026).

**Figure 4:**
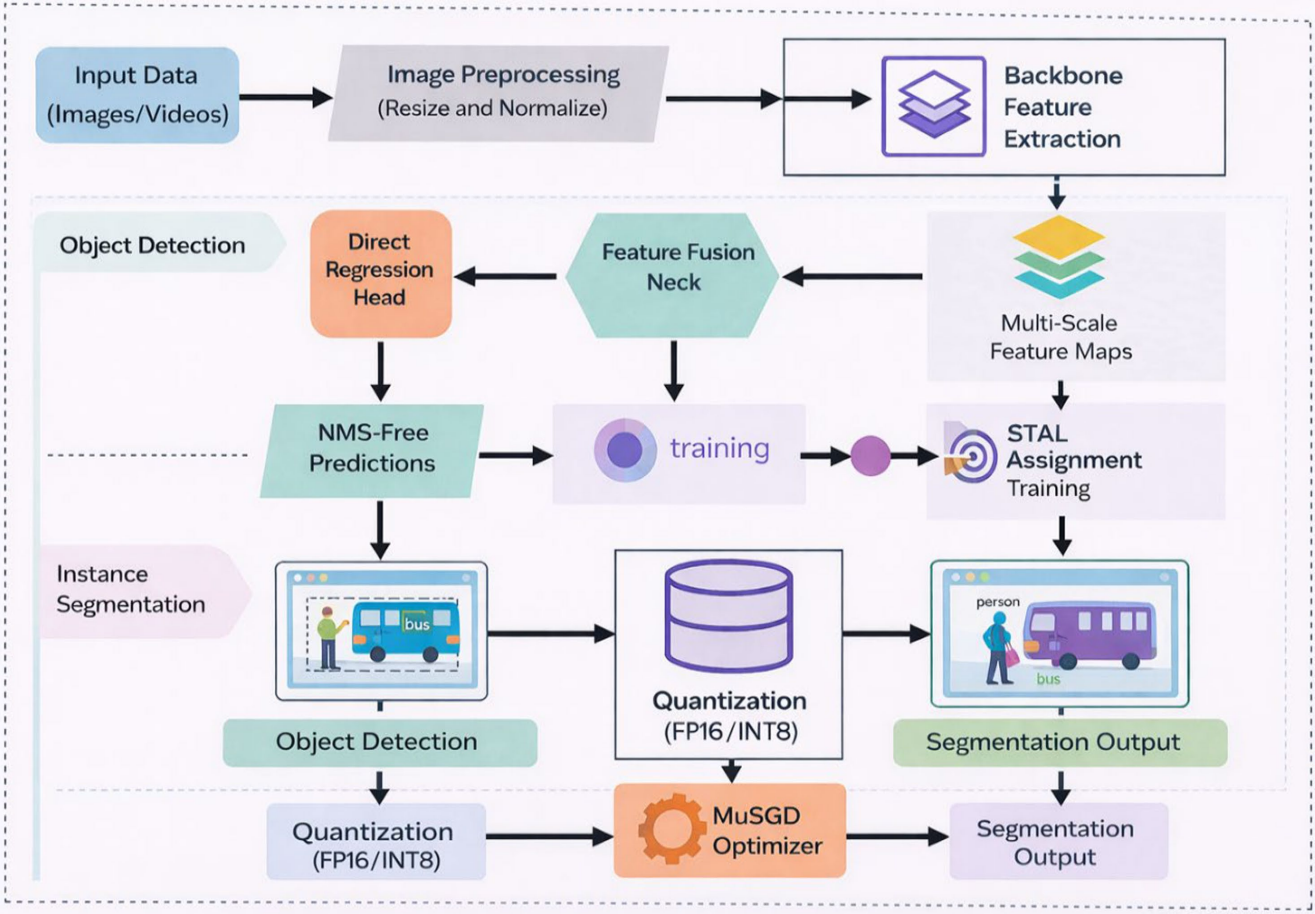
Architecture of YOLO26-SEG network.

In contrast, YOLO26-SEG introduces structural and training-level improvements with a strong emphasis on real-time embedded deployment, as shown in Figure 4. Similar to YOLO11-SEG, the backbone extracts hierarchical features and the neck performs multi-scale fusion across different resolution levels. However, YOLO26-SEG adopts a fully end-to-end, NMS-free detection paradigm, replacing traditional Non-Maximum Suppression post-processing with a direct regression head that outputs final predictions. This design significantly reduces inference latency and simplifies the deployment pipeline, especially on CPU-based or low-power edge platforms. Training stability and small-object sensitivity are enhanced through the Progressive Loss (ProgLoss) function and the Small-Target-Aware Label Assignment (STAL) mechanism, which improve supervision quality for thin and small road elements (Hidayatullah and Tubagus, 2026; Chaman et al., 2025b).

For instance segmentation, YOLO26-SEG integrates a dedicated segmentation branch that generates pixel-level masks using multi-scale mask prototypes and an improved semantic loss formulation. Feature sharing between detection and segmentation tasks minimizes additional computational overhead while maintaining precise mask boundaries and spatial coherence. Furthermore, YOLO26-SEG employs the MuSGD optimizer to accelerate convergence and improve generalization performance. The architecture is compatible with mixed-precision and quantized inference formats such as FP16 and INT8, facilitating hardware-aware optimization on embedded platforms (Sapkota and Karkee, 2026).

Overall, both YOLO11-SEG and YOLO26-SEG implement unified detection and instance segmentation within a single-stage framework. YOLO11-SEG provides a balanced accuracy–complexity trade-off suitable for real-time ADAS perception, while YOLO26-SEG emphasizes inference efficiency, deployment simplicity, and energy-aware optimization. Their comparative integration within the EdgeLane-SEG framework enables a comprehensive evaluation of performance, scalability, and embedded feasibility for safety-critical intelligent transportation systems.

### 2.3 Dataset

To train and evaluate the proposed YOLO11-SEG and YOLO26-SEG models for real-time road-marking detection and instance segmentation, a dedicated dataset comprising 10,542 annotated images and 23,420 labeled object instances was developed. The dataset was specifically designed to support fine-grained, instance-level segmentation of road-surface elements that are critical for Advanced Driver Assistance Systems (ADAS) and autonomous driving perception tasks.

Several public road-scene datasets are available for autonomous driving and ADAS research, such as TuSimple, CULane, BDD100K, ApolloScape, and Mapillary Vistas. However, most of these datasets mainly focus on lane detection, object detection, semantic segmentation, or general road-scene understanding. In contrast, the dataset used in this study is specifically designed for instance-level segmentation of multiple road-surface elements, including lane boundaries, directional arrows, and pedestrian crossings. Therefore, it is more suitable for evaluating the proposed EdgeLane-SEG framework, which requires both object-level localization and pixel-level mask prediction for road marking perception.

**Figure 5:**
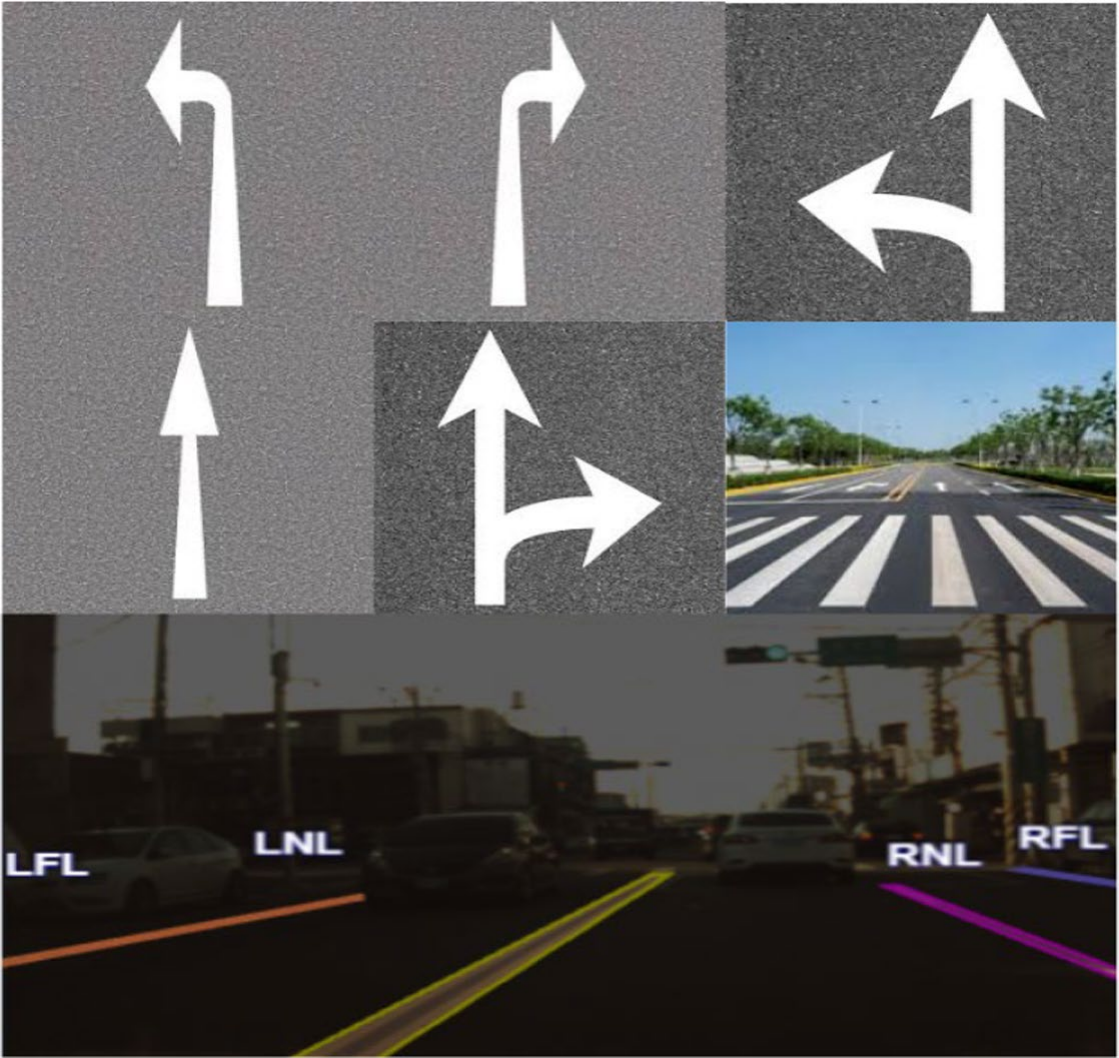
Defined classes of the proposed road marking dataset.

The dataset defines 10 traffic-related classes essential for accurate lateral positioning, lane boundary recognition, and scene understanding. These classes include lane markings—Left Far Lane (LFL), Left Near Lane (LNL), Right Far Lane (RFL), and Right Near Lane (RNL)—as well as directional arrows—Left Arrow (LA), Right Arrow (RA), Straight Arrow (SA), Straight-Left Arrow (SLA), and Straight-Right Arrow (SRA). Additionally, Pedestrian Crossings (PC) are incorporated due to their safety-critical role in intelligent transportation systems. The selected classes exhibit structural similarities and fine geometric variations, particularly among near–far lane pairs and arrow subtypes, introducing controlled classification complexity and enhancing the discriminative learning capability of the models. Representative examples of these defined classes are illustrated in Figure 5.

Images were captured using a forward-facing high-definition camera mounted on a vehicle windshield to replicate real-world ADAS deployment conditions. Data collection encompassed diverse driving environments, including urban intersections, multilane highways, and suburban streets. The dataset incorporates variations in illumination (bright daylight, shadowed regions), road-surface textures (newly painted versus worn markings), perspective distortions, and partial occlusions caused by surrounding vehicles or environmental elements. This diversity strengthens the generalization capability of the trained models under dynamic driving conditions.

All images were manually annotated at the instance level, generating both bounding box coordinates and pixel-wise segmentation masks. The annotation format follows the YOLO segmentation standard, where each object is represented by a class label, normalized bounding box parameters, and polygon-based mask coordinates. This unified structure ensures full compatibility with YOLO11-SEG and YOLO26-SEG architectures and supports joint optimization of detection and segmentation objectives during training.

A stratified data-splitting strategy was applied to maintain balanced class distributions across subsets. Specifically, 70% of the dataset was allocated for training, 20% for validation, and 10% for testing. The validation subset was used for hyperparameter tuning and model selection, whereas the testing subset was reserved exclusively for final performance evaluation. This structured partitioning guarantees reliable generalization assessment and prevents data leakage.

### 2.4 Experimental training strategy

The training strategy of the proposed dual-model framework is designed to ensure stable convergence, fair comparison between YOLO11-SEG and YOLO26-SEG, and compatibility with real-time embedded deployment. Both models are trained under identical experimental conditions to guarantee reproducibility and objective benchmarking.

All input images are resized to 640 × 640 pixels, which represents an optimal trade-off between spatial resolution and computational efficiency. This resolution preserves fine structural details of thin lane markings and road features while maintaining feasibility for real-time inference. Pixel values are normalized to the [0,1] range, and data augmentation techniques including horizontal flipping, slight rotation, brightness variation, and geometric perturbations are applied to enhance generalization under diverse road and lighting conditions.

The training process is conducted using a supervised learning paradigm with instance-level annotations. Optimization is performed using Stochastic Gradient Descent (SGD) with momentum to ensure stable gradient updates and improved convergence behavior. The learning rate scheduling strategy maintains stable training dynamics across epochs. Early stopping is applied if validation metrics plateau, preventing overfitting and reducing unnecessary computational cost.

To ensure reproducibility and address the hardware requirements of the training process, Table 1 summarizes both the hardware–software environment and the training hyperparameters used for YOLO11-SEG and YOLO26-SEG. The same configuration was adopted for both models to guarantee a fair comparison under identical experimental conditions.

**Table 1:** Hardware–software environment and training hyperparameters.

Hardware and Software Environment	Training Hyperparameters
CPU: AMD Ryzen 9 7940HX	Number of epochs: 100
GPU: NVIDIA GeForce RTX 4070	Batch size: 16
GPU memory: 8 GB VRAM	Input image size: 640 × 640
System memory: 32 GB DDR5	Optimizer: SGD
Operating system: Windows 11	Momentum: 0.937
Python version: 3.12.4	Weight decay: 0.0005
PyTorch version: 2.5.1	Initial learning rate: 0.01
CUDA version: 11.8	Learning-rate scheduler: enabled

The proposed dual-model framework optimizes object detection and instance segmentation through a unified multi-task learning objective. Since both YOLO11-SEG and YOLO26-SEG simultaneously predict bounding boxes, confidence scores, class probabilities, and instance masks, a joint loss function is required to ensure balanced optimization of localization, classification, and pixel-level segmentation. This joint formulation is particularly important for road marking perception, where the target objects are often thin, elongated, low-contrast, and partially occluded. By combining detection and segmentation losses, the model learns both object-level localization and fine mask boundaries in a single end-to-end training process. The total multi-task loss is defined in Equation 1:


$$L_{\text{total}}=L_{\det}+\lambda_{\mathrm{seg}}L_{\mathrm{seg}}$$ (1)

Where $L_{\det}$ represents the detection loss, $L_{\mathrm{seg}}$ denotes the segmentation loss, and $\lambda_{\mathrm{seg}}$ is a weighting coefficient balancing detection and mask learning.

The detection loss is defined in Equation 2 and consists of bounding box regression, objectness estimation, and classification:


$$L_{\det}=\lambda_{\mathrm{box}}L_{\mathrm{box}}+\lambda_{\mathrm{obj}}L_{\mathrm{obj}}+\lambda_{\mathrm{cls}}L_{\mathrm{cls}}$$ (2)

The bounding box regression component is defined in Equation 3 and is based on Complete Intersection over Union (CIoU):


$$L_{\mathrm{box}}=1-\text{CIoU}(B_{\text{pred}},B_{\mathrm{gt}})$$ (3)

This formulation accounts for overlap area, center distance, and aspect ratio consistency, improving localization stability for elongated structures such as lane lines.

The objectness loss is computed using Binary Cross-Entropy (BCE), as shown in Equation 4:


$$L_{\mathrm{obj}}=-\sum [y_{\mathrm{obj}}\log(p_{\mathrm{obj}})+(1-y_{\mathrm{obj}})\log(1-p_{\mathrm{obj}})]$$ (4)

The classification loss is also computed using BCE, as shown in Equation 5:


$$L_{\mathrm{cls}}=-\sum_{i=1}^{C}y_{i}\log(p_{i})$$ (5)

Where C is the number of defined road-surface classes.

In parallel, the segmentation branch optimizes mask prediction using the hybrid loss defined in Equation 6, combining BCE and Dice loss:


$$L_{\mathrm{seg}}=L_{\mathrm{BCE}}+L_{\text{Dice}}$$ (6)

The pixel-wise BCE term is defined in Equation 7 and ensures accurate foreground-background classification:


$$L_{\mathrm{BCE}}=-\frac{1}{N}\sum_{j=1}^{N}[y_{j}\log(p_{j})+(1-y_{j})\log(1-p_{j})]$$ (7)

The Dice loss is defined in Equation 8 and maximizes mask overlap:


$$L_{\text{Dice}}=1-\frac{2\sum p_{j}y_{j}+\epsilon }{\sum p_{j}+\sum y_{j}+\epsilon }$$ (8)

The final optimization objective is defined in Equation 9:


$$L_{\text{total}}=\lambda_{\mathrm{box}}L_{\mathrm{box}}+\lambda_{\mathrm{obj}}L_{\mathrm{obj}}+\lambda_{\mathrm{cls}}L_{\mathrm{cls}}+\lambda_{\mathrm{seg}}(L_{\mathrm{BCE}}+L_{\text{Dice}})$$ (9)

This unified loss function allows the models to jointly learn accurate bounding box localization, reliable class prediction, and precise instance mask generation. The use of BCE improves pixel-level foreground discrimination, while Dice loss reduces the effect of class imbalance between thin road markings and the dominant road background. Therefore, the joint loss formulation is well suited for road marking and lane line instance segmentation, where accurate mask continuity and boundary precision are essential for ADAS applications.

Validation monitoring is conducted after each epoch using Precision, Recall, F1-score, mAP@0.5, and mAP@0.5–0.95. The best-performing model weights are automatically saved according to validation mAP performance, while periodic checkpoints are stored to ensure training recovery and reproducibility. This training configuration ensures stable convergence, balanced multi-task optimization, and computational efficiency, preparing the models for optimized deployment on embedded edge platforms such as Jetson Nano, Raspberry Pi 5, Intel VPU, and Hailo-8 NPU.

### 2.5 Embedded deployment and hardware-aware optimization

**Figure 6:**
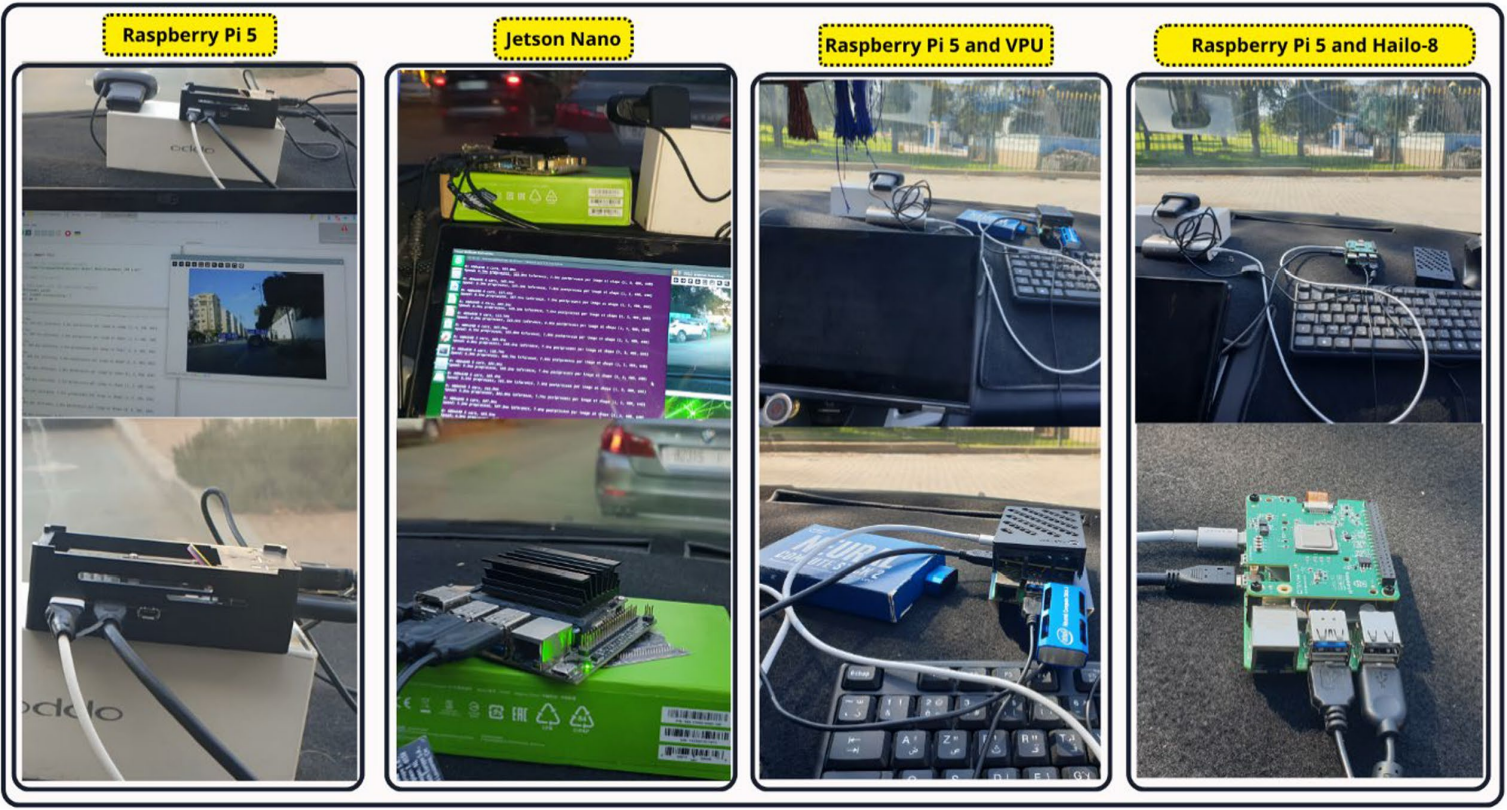
Experimental setup for EdgeSafe-Vision deployment on embedded platforms.

The transition from high-performance GPU-based training to real-time embedded deployment requires a structured model conversion and hardware-aware optimization pipeline. In this work, both YOLO11-SEG and YOLO26-SEG were converted and optimized across heterogeneous edge platforms to ensure minimal latency, stable throughput, and energy-efficient operation under ADAS constraints. The complete embedded deployment architecture, including camera integration, processing pipeline, and hardware configuration inside the vehicle, is illustrated in Figure 6.

After training in PyTorch, the models were exported to the ONNX (Open Neural Network Exchange) format to establish a hardware-independent computational graph. During export, inference-specific configurations were applied, including fixed input resolution of 640 × 640, batch size equal to one, and removal of training-only operators. Static graph generation enabled more aggressive optimization by downstream inference engines. Graph simplification techniques were applied to eliminate redundant nodes and fuse compatible layers, improving runtime efficiency.

A key contribution of this deployment pipeline lies in its hardware-aware optimization strategy rather than a simple transfer of trained models across platforms. Each target device was assigned a dedicated inference backend and precision configuration according to its computational architecture. This strategy enables a systematic comparison of GPU-, CPU-, VPU-, and NPU-based acceleration in terms of inference latency, throughput, power consumption, and performance-per-watt efficiency.

To further clarify the proposed hardware-aware deployment strategy, Table 2 summarizes the inference backend, optimization techniques, and precision format adopted for each embedded platform. This table highlights that the proposed deployment is not limited to running the models on different devices, but applies platform-specific optimization according to the computational architecture of each target system.

**Table 2:** Hardware-aware optimization strategy adopted for embedded deployment.

Platform	Backend	Optimization	Precision
Jetson Nano	TensorRT	Layer fusion, kernel tuning, GPU memory optimization	FP16/INT8
Raspberry Pi 5	ONNX Runtime	Graph optimization, operator fusion, multi-threading	FP32
Raspberry Pi 5 + Intel VPU	OpenVINO IR	MYRIAD plugin, CPU–VPU execution	FP16
Raspberry Pi 5 + Hailo-8 NPU	HailoRT/HEF	INT8 quantization, graph partitioning, kernel fusion	INT8

For the NVIDIA Jetson Nano, the ONNX models were converted into TensorRT engines. TensorRT performs graph optimization, kernel auto-tuning, tensor fusion, and memory layout restructuring specifically tailored to NVIDIA GPU architectures. FP16 precision mode was employed to reduce memory bandwidth and accelerate inference while maintaining segmentation accuracy. Optional INT8 quantization was evaluated using representative calibration datasets to further reduce latency and power consumption. GPU-accelerated preprocessing using CUDA minimized unnecessary CPU–GPU memory transfers, and memory buffers were pre-allocated to ensure deterministic runtime behavior (Hakani and Rawat, 2024).

For the Raspberry Pi 5, inference was executed using ONNX Runtime configured for optimized CPU-based deployment. Full graph optimization was enabled, including operator fusion and memory reuse strategies. Multi-threading through OpenMP was configured to maximize utilization of ARM Cortex-A76 cores while maintaining thermal stability. NEON SIMD acceleration was leveraged where supported to enhance arithmetic throughput. This configuration ensured efficient inference under constrained power conditions (Raspberry Pi Hardware–Raspberry Pi Documentation, 2025).

For the VPU-assisted configuration, the ONNX models were converted to OpenVINO Intermediate Representation (IR) format with FP16 precision and fixed input resolution. The OpenVINO Inference Engine executed the model using the MYRIAD plugin, enabling the Intel Movidius Myriad X VPU to process compute-intensive convolutional layers while the CPU handled preprocessing and post-processing. This heterogeneous architecture significantly reduced CPU workload and improved energy efficiency (Mohamed et al., 2023).

For the NPU-assisted configuration, the Raspberry Pi 5 was integrated with the Hailo-8 Neural Processing Unit (NPU) via the PCIe-based Hailo AI Hat. The Hailo-8 represents a next-generation edge AI accelerator, delivering up to 26 TOPS within a low-power envelope of approximately 2.5–3 W, corresponding to an efficiency exceeding 8 TOPS/W. This makes it particularly suitable for real-time embedded ADAS applications requiring high throughput and energy efficiency.

The deployment environment consisted of Raspberry Pi OS (Bookworm), Python 3.11.2, HailoRT 4.21.0, OpenCV 4.11.0, and GStreamer 1.22.0 for real-time video synchronization. The trained YOLO11-SEG and YOLO26-SEG models were exported to ONNX format and quantized to INT8 precision before being compiled using the Hailo Dataflow Compiler. During compilation, advanced optimizations such as graph partitioning, kernel fusion, and memory-efficient scheduling were applied, resulting in a Hailo Executable File (HEF) optimized for NPU execution.

During inference, the Hailo-8 NPU handled all compute-intensive neural network operations, while the Raspberry Pi CPU was responsible for preprocessing, post-processing, and visualization. This heterogeneous execution model significantly reduced CPU load and improved overall system efficiency. Experimental results demonstrated that the Hailo-8 configuration achieved the highest performance-per-watt (FPS/W) among all evaluated platforms, highlighting the advantages of dedicated NPU acceleration for real-time edge AI deploymen (Bueno et al., 2025).

### 2.6 Accuracy and embedded performance evaluation

The performance of the proposed EdgeLane-SEG framework is evaluated using a comprehensive set of metrics that jointly assess object detection accuracy, instance segmentation quality, and embedded real-time feasibility. Since the system performs multi-task learning for both bounding box localization and pixel-level mask prediction, evaluation metrics are selected to reflect spatial overlap accuracy, classification reliability, and computational efficiency under deployment constraints.

For object detection, the primary evaluation criterion is the Intersection over Union (IoU), which quantifies the overlap between predicted and ground-truth bounding boxes. A detection is considered correct when the IoU exceeds a predefined threshold. In parallel, instance segmentation quality is assessed using mask-level IoU, computed on pixel-wise overlap between predicted and ground-truth masks. These overlap measures serve as the foundation for computing Average Precision (AP) and mean Average Precision (mAP), which summarize detection and segmentation performance across recall levels and object classes. Two evaluation settings are considered: mAP@0.5, using a fixed IoU threshold of 0.5, and mAP@0.5:0.95, which averages AP over multiple IoU thresholds from 0.5 to 0.95. The latter provides a stricter and more comprehensive assessment of localization and boundary precision (Zhenbo et al., 2026; Zeng et al., 2025).

Detection reliability is further evaluated using Precision, Recall, and F1-score. Precision measures the proportion of correctly predicted objects among all positive predictions, while Recall quantifies the proportion of detected objects among all ground-truth instances. The F1-score provides a harmonic balance between these two metrics, ensuring that both false positives and false negatives are considered. These metrics are particularly important for thin and elongated structures such as lane markings, where minor localization errors can significantly affect performance (Sanikommu et al., 2025).

Beyond accuracy evaluation, runtime performance is measured to assess embedded feasibility. Inference latency is computed as the average processing time per frame, and throughput is expressed in Frames Per Second (FPS) (Barua et al., 2026). Energy efficiency is evaluated using FPS per Watt (FPS/W), which reflects performance normalized by power consumption. These deployment-oriented indicators are essential for ADAS applications operating under strict energy and real-time constraints.

The mathematical formulations of the adopted metrics are summarized in Equations 10–15:


$$\mathrm{IoU}=\frac{|B_{\text{pred}}\cap B_{\mathrm{gt}}|}{|B_{\text{pred}}\cup B_{\mathrm{gt}}|}$$ (10)

$$\text{Precision}=\frac{\mathrm{TP}}{\mathrm{TP}+\mathrm{FP}}$$ (11)

$$\text{Recall}=\frac{\mathrm{TP}}{\mathrm{TP}+\mathrm{FN}}$$ (12)

$$F1=2\times \frac{\text{Precision}\times \text{Recall}}{\text{Precision}+\text{Recall}}$$ (13)

$$\mathrm{mAP}=\frac{1}{C}\sum_{i=1}^{C}{\mathrm{AP}}_{i}$$ (14)

$$\mathrm{FPS}=\frac{1}{T_{\inf}}$$ (15)

Equations 10–15 define IoU, Precision, Recall, F1-score, mAP, and FPS, respectively. Where TP , FP , and FN denote true positives, false positives, and false negatives; C represents the number of classes; $T_{\inf}$ is the average inference time per frame; and P is the average power consumption.

Together, these metrics provide a rigorous and multi-dimensional evaluation of detection accuracy, segmentation quality, inference speed, and energy efficiency, ensuring that both YOLO11-SEG and YOLO26-SEG are assessed under realistic embedded ADAS deployment conditions.

## 3 Results

The experimental evaluation demonstrates the strong robustness, convergence stability, and segmentation accuracy of both YOLO26-SEG and YOLO11-SEG models for real-time road marking and lane line instance segmentation in ADAS scenarios. Training was conducted using PyTorch v2.5.1 with NVIDIA GPU acceleration enabled via CUDA 11.8 for 100 epochs to ensure sufficient convergence.

The optimization process relied on Stochastic Gradient Descent (SGD) with an initial learning rate of 0.01, momentum of 0.937, and scheduled learning-rate decay to promote smooth and stable training dynamics. L2 regularization with a weight decay of 0.0005 was applied to reduce overfitting and improve generalization. Experiments were executed on a high-performance workstation equipped with an AMD Ryzen 9 7940HX CPU, NVIDIA GeForce RTX 4070 GPU with 8 GB VRAM, and 32 GB DDR5 RAM under Windows 11.

**Figure 7:**
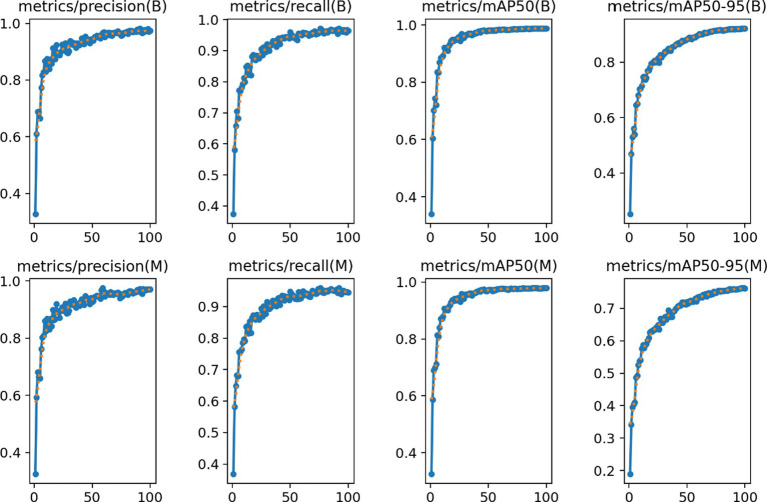
YOLO26-SEG training metrics.

**Figure 8:**
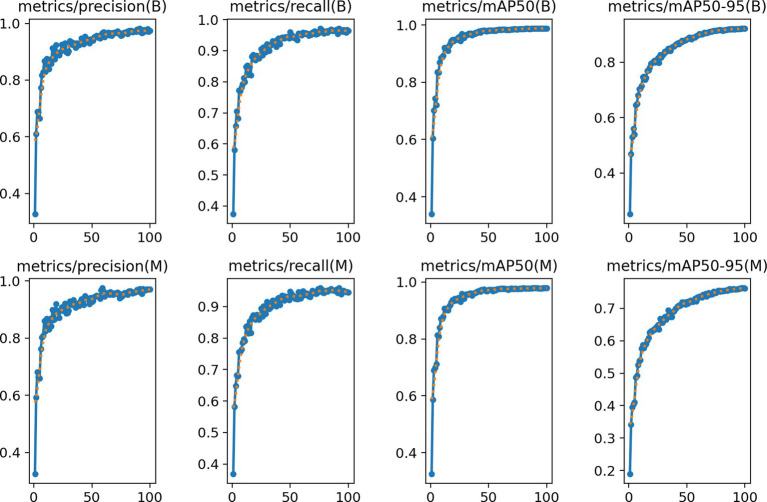
YOLO26-SEG performance metrics.

**Figure 9:**
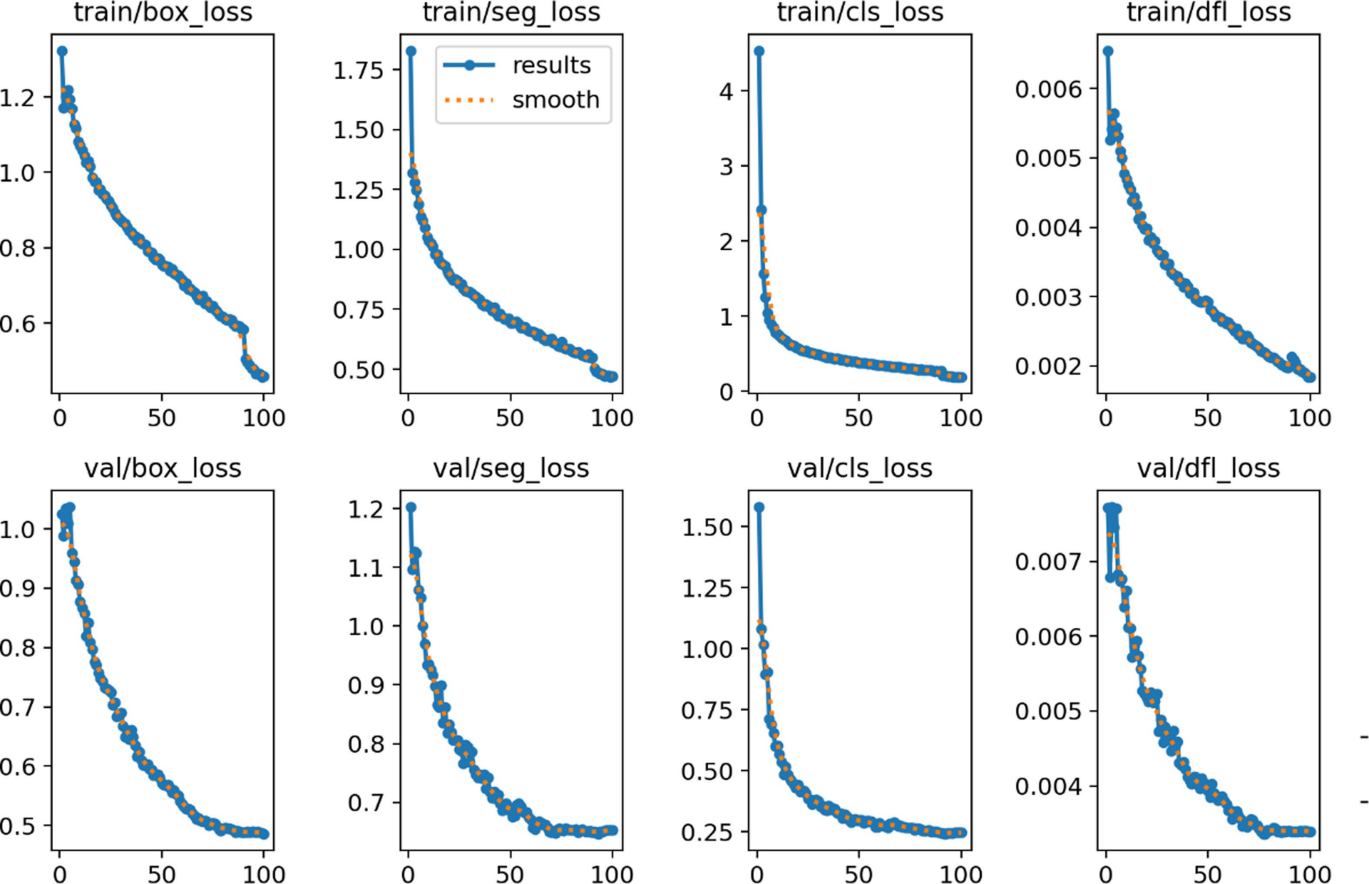
YOLO11-SEG training metrics.

**Figure 10:**
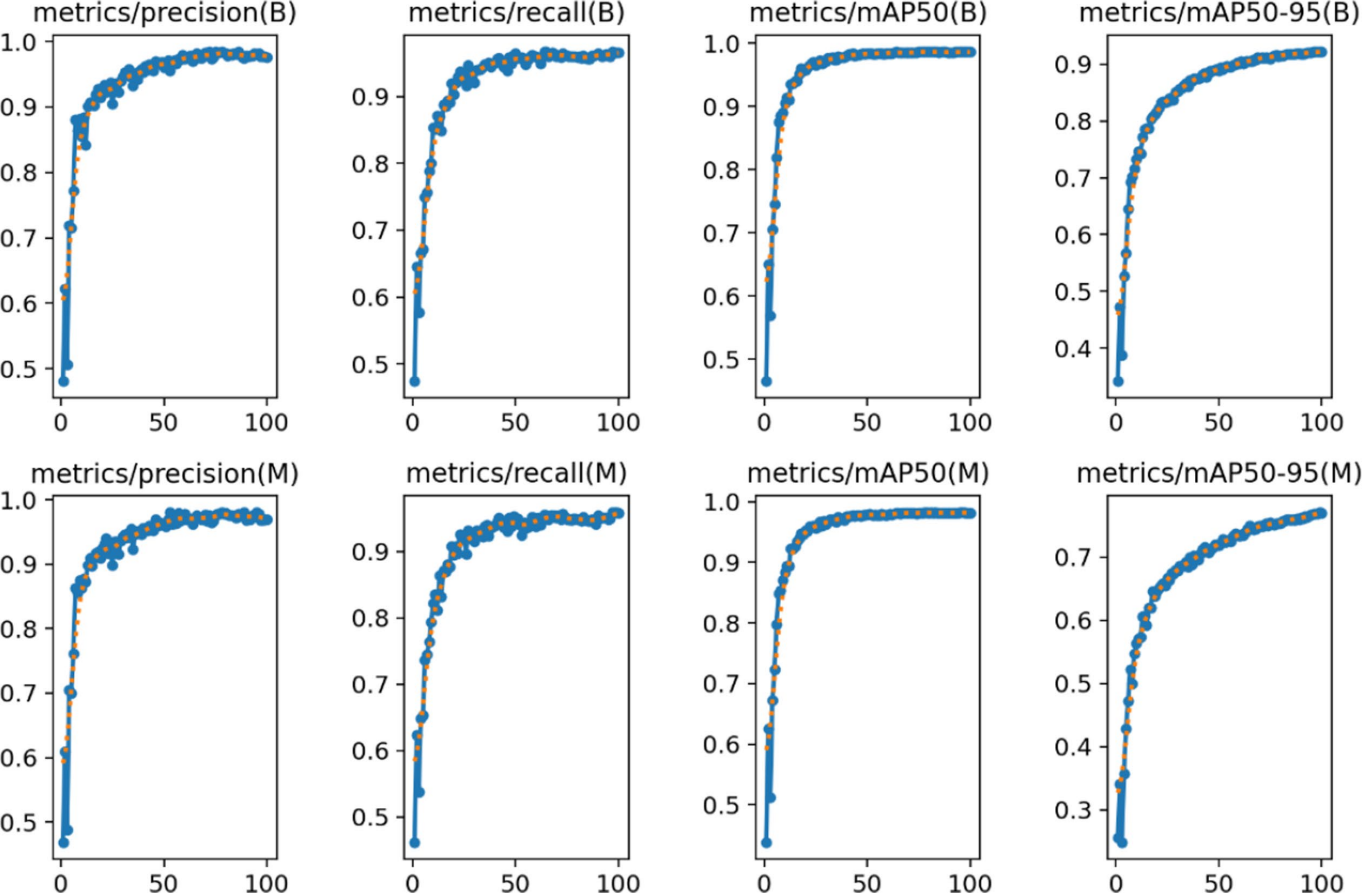
YOLO11-SEG performance metrics.

As illustrated in Figures 7–10, YOLO26-SEG and YOLO11-SEG exhibit consistent convergence behavior. Training and validation losses, including box, classification, DFL, and segmentation losses, decrease progressively and stabilize toward the final epochs, indicating effective optimization and minimal overfitting. Precision, recall, mAP@0.5, and mAP@0.5–0.95 curves show rapid improvement during the early epochs followed by stable saturation, confirming reliable generalization.

Quantitative performance comparison is summarized in Table 3. YOLOv8-SEG was included only as a training and validation benchmark to compare the accuracy of YOLO11-SEG and YOLO26-SEG with a widely used YOLO-based segmentation baseline. All three models were trained and evaluated using the same dataset, input resolution, and accuracy metrics.

**Table 3:** Performance evaluation of the YOLO models.

Metrics	YOLO26-SEG model		YOLO11-SEG model		YOLOv8-SEG model	
	Box	Mask	Box	Mask	Box	Mask
Precision	98.2%	97.4%	97.4%	96.5%	96.3%	96.1%
Recall	95.8%	94.9%	95.1%	95.6%	94.1%	94.8%
F1-score	97%	96.1%	96.2%	95.8%	95.2%	95.4%
mAP@0.5	98.8%	97.9%	98.4%	98.2%	97.9%	98%
mAP@0.50.95	92.2%	76.2%	92.3%	77.1%	92%	75.3%

YOLO26-SEG achieves the highest box precision of 98.2% and mask precision of 97.4%, outperforming YOLO11-SEG and YOLOv8-SEG in terms of precision. For box-level detection, YOLO26-SEG also obtains the highest recall, F1-score, and mAP@0.5, reaching 95.8, 97.0, and 98.8%, respectively. For instance segmentation, YOLO26-SEG provides strong mask-level performance, with 97.4% precision, 94.9% recall, 96.1% F1-score, and 97.9% mAP@0.5.

Although YOLO11-SEG achieves a slightly higher mask mAP@0.5–0.95 of 77.1% compared with 76.2% for YOLO26-SEG, the difference remains limited. YOLOv8-SEG also provides competitive performance, especially for mask mAP@0.5, but it remains slightly lower than YOLO26-SEG in most box-level metrics, including precision, recall, F1-score, and mAP@0.5. Overall, YOLO26-SEG offers the best balance between detection accuracy, segmentation performance, and deployment suitability within the proposed EdgeLane-SEG framework. Therefore, the embedded deployment stage focuses on YOLO11-SEG and YOLO26-SEG, with YOLO26-SEG selected as the most suitable model for real-time embedded ADAS deployment.

To improve the readability of the Results section, the detailed class-wise precision–recall curves were not emphasized in the main analysis. Instead, the evaluation focuses on the quantitative metrics reported in Table 3, including Precision, Recall, F1-score, mAP@0.5, and mAP@0.5–0.95 for both box detection and mask segmentation. These metrics provide a more compact and directly comparable assessment of YOLO26-SEG, YOLO11-SEG, and the YOLOv8-SEG benchmark.

**Figure 11:**
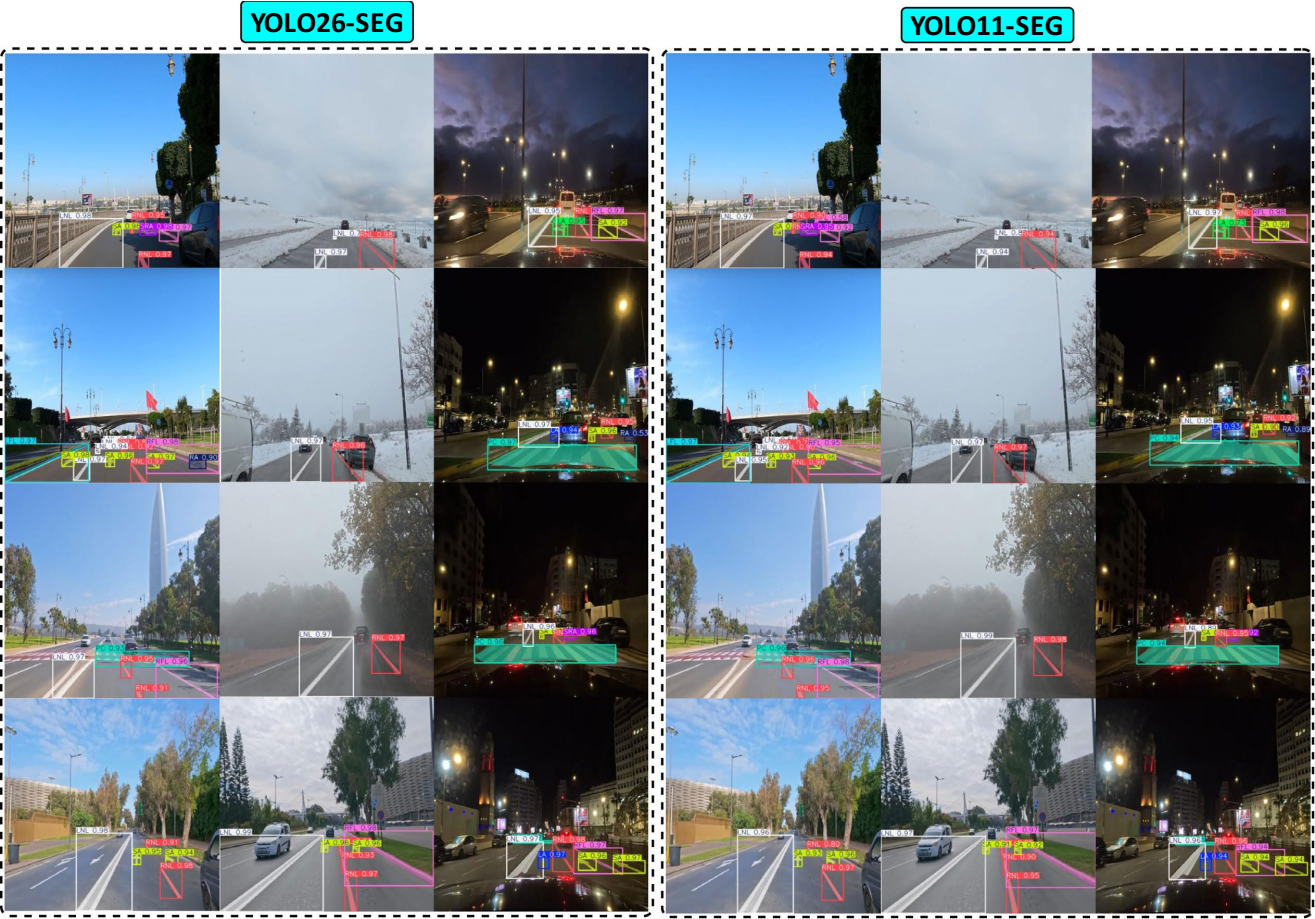
Comparative qualitative results of YOLO26-SEG and YOLO11-SEG.

To evaluate the real-world embedded performance of the proposed EdgeLane-SEG framework, both YOLO26-SEG and YOLO11-SEG were deployed and tested on four representative edge platforms: NVIDIA Jetson Nano, Raspberry Pi 5, Raspberry Pi 5 integrated with an Intel Movidius VPU accelerator, and Raspberry Pi 5 combined with a Hailo-8 NPU accelerator. As illustrated in Figure 11, qualitative results confirm that both models consistently achieve accurate object detection and instance segmentation of key road-surface classes, including lane boundaries (LFL, LNL, RFL, RNL), directional arrows (LA, RA, SA, SLA, SRA), and pedestrian crossings (PC), under highly diverse driving conditions.

The evaluated scenarios cover clear daylight, dense urban intersections, multilane highways, suburban streets, nighttime illumination, foggy environments, snow-covered roads, and partial occlusions caused by vehicles and environmental elements. Despite these variations, both architectures demonstrate strong spatial consistency and stable confidence predictions. In particular, YOLO26-SEG exhibits slightly improved segmentation smoothness and structural continuity in elongated lane markings, especially in distant regions and low-contrast conditions such as fog and nighttime scenes. YOLO11-SEG maintains comparable detection performance, although minor boundary irregularities can occasionally be observed under reduced visibility.

Across all embedded platforms, inference results remain visually stable and temporally consistent, confirming the suitability of the framework for real-time deployment in resource-constrained ADAS environments. The qualitative consistency across environmental variations further validates the robustness, adaptability, and generalization capability of the proposed dual-model segmentation approach.

A comprehensive comparison of hardware specifications, inference frameworks, computational performance, energy efficiency, and cost was conducted across five processing platforms used to evaluate YOLO11-SEG and YOLO26-SEG, as summarized in Table 4. The investigated systems include a high-performance personal computer, NVIDIA Jetson Nano, Raspberry Pi 5, Raspberry Pi 5 integrated with an Intel Movidius Myriad X VPU accelerator, and Raspberry Pi 5 combined with a Hailo-8 NPU accelerator. The objective of this analysis is to identify the most suitable platform for real-time embedded ADAS deployment under performance, power, and cost constraints.

**Table 4:** Comparative hardware specifications, inference performance, energy efficiency, and cost analysis of YOLO11-SEG and YOLO26-SEG across desktop and embedded platforms.

Processing system		Personal computer	NVIDIA Jetson Nano	Raspberry Pi 5	Raspberry Pi 5 + Intel Movidius VPU	Raspberry Pi 5 + Hailo-8 NPU
CPU		AMD Ryzen 9 7940HX	Quad-core ARM Cortex-A57	Broadcom BCM2712, quad-core ARM Cortex-A76 @ 2.4 GHz	Broadcom BCM2712, quad-core ARM Cortex-A76 @ 2.4 GHz	Broadcom BCM2712, quad-core ARM Cortex-A76 @ 2.4 GHz
GPU / Accelerator		NVIDIA GeForce RTX 4070, 8 GB VRAM	128-core Maxwell GPU	VideoCore VII GPU	Intel Movidius Myriad X VPU, 1 TOPS	Hailo-8 NPU, 26 TOPS INT8
YOLO11-SEG	Power	115 W	10-11W	5-7W	5-6W	3–3.5 W
	Latency	19 ms	110 ms	420 ms	320 ms	23 ms
	FPS	52.6 FPS	9 FPS	2.38 FPS	3.1 FPS	43.5 FPS
	Energy efficiency	0.46 FPS/W	0.87 FPS/W	0.40 FPS/W	0.56 FPS/W	13.45 FPS/W
YOLO26-SEG	Power	115W	9–10 W	5-6W	4–5 W	2.5–3 W
	Latency	12 ms	100 ms	340 ms	290 ms	19-20ms
	FPS	83.3 FPS	10 FPS	2.9 FPS	3.45 FPS	50–52.6 FPS
	Energy efficiency	0.72 FPS/W	1.05 FPS/W	0.53 FPS/W	0.77 FPS/W	18.85 FPS/W
Estimated cost		$2,482.00	$350.00	$220.00	$400.00	$420

The personal computer, powered by an AMD Ryzen 9 7940HX CPU and NVIDIA RTX 4070 GPU, achieves the highest raw inference throughput, reaching 52.6 FPS for YOLO11-SEG and 83.3 FPS for YOLO26-SEG. However, its high-power consumption of 115 W results in moderate energy efficiencies of 0.46 FPS/W and 0.72 FPS/W, respectively. Therefore, despite its superior computational capability, the desktop platform is less suitable for in-vehicle embedded deployment due to its high energy demand and cost.

The NVIDIA Jetson Nano provides a balanced embedded GPU-based solution. Using TensorRT acceleration with FP16/INT8 optimization, it achieves 9 FPS for YOLO11-SEG and 10 FPS for YOLO26-SEG, with energy efficiencies of 0.87 FPS/W and 1.05 FPS/W, respectively. This demonstrates a reasonable trade-off between inference speed, power consumption, and affordability.

The Raspberry Pi 5 with CPU-based ONNX Runtime inference shows limited real-time capability due to the absence of dedicated AI acceleration. It achieves 2.38 FPS for YOLO11-SEG and 2.9 FPS for YOLO26-SEG, with efficiencies of 0.40 FPS/W and 0.53 FPS/W, respectively. However, its low cost and modest power draw make it useful for budget-constrained applications with relaxed latency requirements.

The Raspberry Pi 5 combined with the Intel Movidius Myriad X VPU improves inference performance compared with CPU-only execution. It achieves 3.1 FPS and 0.56 FPS/W for YOLO11-SEG, and 3.45 FPS and 0.77 FPS/W for YOLO26-SEG. This confirms the benefit of lightweight VPU acceleration, although the achieved throughput remains below strict real-time ADAS requirements.

The Raspberry Pi 5 with Hailo-8 NPU achieves the best embedded performance and energy efficiency among the evaluated edge platforms. With INT8 acceleration using HailoRT and HEF deployment, YOLO11-SEG reaches 43.5 FPS with a mean efficiency of 13.45 FPS/W, while YOLO26-SEG achieves 50–52.6 FPS with a mean efficiency of 18.85 FPS/W. These results show that dedicated NPU acceleration provides a major improvement in both throughput and performance-per-watt, making this configuration the most suitable low-power platform for real-time embedded ADAS perception.

YOLO26-SEG consistently surpasses YOLO11-SEG across all platforms in inference speed and energy efficiency. Among embedded solutions, the Raspberry Pi 5 + Hailo-8 NPU provides the best performance-per-watt ratio and satisfies real-time requirements with low power consumption. The Jetson Nano offers a balanced GPU-based alternative, while the Raspberry Pi 5 + VPU provides a modest improvement over CPU-only inference. These findings confirm the practical suitability of the proposed EdgeLane-SEG framework for real-time deployment in resource-constrained embedded ADAS systems, where latency, power efficiency, and cost are decisive design parameters.

## 4 Discussion

The experimental results demonstrate that the proposed EdgeLane-SEG framework achieves a robust balance between detection accuracy, segmentation precision, computational efficiency, and embedded deployability. The comparative evaluation between YOLO11-SEG and YOLO26-SEG reveals consistent architectural improvements in YOLO26-SEG, particularly in inference speed, optimization efficiency, and hardware adaptability.

From an architectural standpoint, YOLO26 introduces several key enhancements that directly contribute to its superior performance on embedded platforms. First, the removal of Distribution Focal Loss (DFL) reduces computational complexity during regression while maintaining localization accuracy. Second, the adoption of an end-to-end NMS-free inference design minimizes post-processing overhead, which is especially beneficial for CPU-based and low-power edge devices. Third, the integration of Progressive Loss (ProgLoss) combined with Small-Target-Aware Label Assignment (STAL) improves feature supervision and enhances detection stability for small or thin objects such as lane markings and directional arrows. These improvements are particularly relevant in road-marking segmentation tasks, where objects are elongated, partially occluded, and geometrically subtle. Additionally, the use of the MuSGD optimizer enhances convergence efficiency and contributes to faster inference throughput without increasing parameter count. Collectively, these refinements explain the consistent FPS and FPS/W improvements observed across the evaluated platforms.

From an accuracy perspective, both models achieve strong box-level detection performance, with mAP@0.5 exceeding 98%. However, YOLO26-SEG demonstrates improved precision stability and better energy-normalized efficiency while maintaining comparable segmentation quality. The stable training and validation loss curves indicate strong generalization capability, particularly due to the combined BCE–Dice segmentation loss, which effectively handles class imbalance between thin foreground markings and dominant background regions.

Deployment benchmarking further confirms the architectural efficiency of YOLO26-SEG. While the high-performance desktop platform maximizes raw throughput, its high-power consumption limits embedded applicability. The Jetson Nano provides a balanced GPU-based embedded alternative, while Raspberry Pi 5 with Intel Movidius VPU improves efficiency compared with CPU-only inference. More importantly, the Raspberry Pi 5 combined with the Hailo-8 NPU achieves the highest performance-per-watt ratio, demonstrating the advantage of dedicated neural acceleration for real-time ADAS perception. Across all platforms, YOLO26-SEG consistently delivers higher FPS and superior FPS/W compared with YOLO11-SEG, validating the computational benefits of its refined architecture.

Despite these strengths, segmentation at high IoU thresholds (mAP@0.5–0.95) remains more challenging than box detection, reflecting the inherent difficulty of precise pixel-level delineation of slender lane structures. Future improvements may include lightweight attention mechanisms, lane-continuity constraints, or temporal smoothing strategies to further enhance mask-level robustness under adverse weather, occlusion, and low-contrast conditions.

Overall, the architectural advancements introduced in YOLO26 significantly improve computational efficiency, convergence stability, and embedded suitability while preserving high detection and segmentation accuracy. These results confirm that YOLO26-SEG represents a deployment-oriented evolution within the YOLO family, making it particularly suitable for real-time embedded ADAS and autonomous driving applications where performance-per-watt, latency, and reliability are critical constraints

## 5 Conclusion

This paper introduced EdgeLane-SEG, an energy-efficient embedded edge artificial intelligence framework for real-time road marking and lane line instance segmentation in Advanced Driver Assistance Systems (ADAS) and autonomous driving environments. The proposed approach integrates two advanced single-stage segmentation architectures, YOLO11-SEG and YOLO26-SEG, within a unified multi-task learning and hardware-aware deployment pipeline. By jointly optimizing bounding box regression, object confidence, classification, and pixel-level mask prediction, the framework ensures accurate localization and precise delineation of thin and elongated road structures such as lane boundaries and directional arrows.

Extensive experimental evaluation demonstrates that both models achieve strong detection and segmentation performance, with detection mAP@0.5 exceeding 98% and stable precision–recall characteristics across all 10 defined road-surface classes. Comparative results show that YOLO26-SEG provides improved inference speed and energy efficiency across all evaluated platforms while maintaining comparable segmentation quality to YOLO11-SEG. Embedded benchmarking confirms the feasibility of real-time deployment on NVIDIA Jetson Nano, Raspberry Pi 5, Raspberry Pi 5 with Intel Movidius VPU, and Raspberry Pi 5 with Hailo-8 NPU. In particular, the Hailo-8 NPU configuration achieves the highest performance-per-watt ratio, confirming the benefit of dedicated neural acceleration for energy-aware ADAS perception.

This study bridges the gap between high-accuracy deep learning segmentation models and practical embedded deployment constraints. By combining robust multi-task optimization, structured dataset design, and platform-specific inference acceleration, EdgeLane-SEG offers a scalable, cost-effective, and power-efficient perception solution for intelligent transportation systems.

Future work will explore lightweight attention mechanisms, structural continuity constraints for lane topology modeling, temporal smoothing, and multi-sensor fusion to further enhance robustness under adverse weather, occlusion, and complex traffic conditions.

## Data Availability

The raw data supporting the conclusions of this article will be made available by the authors, without undue reservation.

## References

[ref2] Aljahani F. H. (2025). RT-DETR edge deployment: real-time detection transformer for distracted driving detection. Int. J. Adv. Comput. Sci. Appl. 16:61094. doi: 10.14569/IJACSA.2025.0161094

[ref3] Alsayaydeh J. A. J. Bacarra R. Ibrahim A. F. B. T. Farid M. Al-Hilali A. Herawan S. G. (2026). DriveRight: an embedded AI-based multi-hazard detection and alert system for safe and sustainable driving. Int. J. Adv. Comput. Sci. Appl. 17:70105. doi: 10.14569/IJACSA.2026.0170105

[ref4] Barua P. Yeahea M. A. F. Rashid R. N. Fahad Yeahea M. Islam M. R. Zaman M. U. . (2026). YOLO-TeethSeg: a resource-efficient approach to multi-class teeth instance segmentation using lightweight YOLO-based models. Biomed. Signal Process. Control 120:109952. doi: 10.1016/j.bspc.2026.109952

[ref5] Biswas A. Wang H.-C. (2023). Autonomous vehicles enabled by the integration of IoT, edge intelligence, 5G, and Blockchain. Sensors 23:4. doi: 10.3390/s23041963, 36850560 PMC9963447

[ref6] Bruno D. R. Berri R. A. Barbosa F. M. Osório F. S. (2023). CARINA project: visual perception systems applied for autonomous vehicles and advanced driver assistance systems (ADAS). IEEE Access 11, 69720–69749. doi: 10.1109/ACCESS.2023.3287491

[ref7] Bueno G. Sanchez-Vargas L. Diaz-Maroto A. Ruiz-Santaquiteria J. Blanco M. Salido J. . (2025). Real-time edge computing vs. GPU-accelerated pipelines for low-cost microscopy applications. Electronics 14:930. doi: 10.3390/electronics14050930

[ref8] Caballero-Ramírez D. Tortorella G. García-Alcaraz J. Jiménez-Macías E. Limon-Romero J. Baez-Lopez Y. . (2025). Deep learning-based detection of wine bottle capsules for contamination prevention. Results Eng. 27:106388. doi: 10.1016/j.rineng.2025.106388, 38826717

[ref9] Chaman M. El Maliki A. Dahou H. Hadjoudja A. (2026a). Benchmarking YOLO-based deep learning models for real-time object detection in hybrid ADAS and intelligent transportation systems. Results Eng. 29:108942. doi: 10.1016/j.rineng.2025.108942

[ref10] Chaman M. El Maliki A Jariri N. Bouyoussef W. Belaaribi A. Yanboiy H. . (2025b). *Detection and Instance Segmentation of road Markings and Lane Lines Using YOLOv11-SEG for ADAS and Autonomous Driving 2025 International Conference on Electrical Systems and Automation (ICESA)*. pp. 1–6.

[ref11] Chaman M. El Maliki A. Jariri N. El Mrabet A. Dahou H. Laamari H. . (2025a). A real-time vehicle detection system for ADAS in autonomous vehicles using YOLOv11 deep neural network on embedded edge platforms. Eng. Technol. Appl. Sci. Res. 15, 28077–28082. doi: 10.48084/etasr.12138

[ref12] Chaman M. El Maliki A. Natij Y. Dahou H. Hadjoudja A. (2026b). Deep learning–based real time speed limit sign detection with YOLOv12 on edge AI platforms for embedded ADAS. Bull. Electr. Eng. Inform. 15, 396–405. doi: 10.11591/eei.v15i1.11128

[ref13] Cheng Q. Ling J. Yang Y. Liu K. Li H. Huang X. (2024). InstLane dataset and geometry-aware network for instance segmentation of lane line detection. Remote Sens. 16:2751. doi: 10.3390/rs16152751

[ref14] Cheng W. Wang X. Mao B. (2023). Research on lane line detection algorithm based on instance segmentation. Sensors 23:789. doi: 10.3390/s23020789, 36679585 PMC9866380

[ref17] Farhani N. Hermassi M. Hajjaji M. A. Zrigui M. (2026). Multi-scale attention and transformer-enhanced YOLO architecture for robust traffic sign detection in complex visual environments. Intell. Artif. 20, 29–52. doi: 10.1177/17248035251390608

[ref18] Feilong Q. Khan N. A. Jhanjhi N. Z. Ashfaq F. Hendrawati T. D. (2025). Improved YOLOv5 lane line real time segmentation system integrating seg head network. Eng. Proc. 107:49. doi: 10.3390/engproc2025107049

[ref20] Gu X. Zhang G. (2025). PSC-YOLO: a lightweight model for urban road instance segmentation. J. Real-Time Image Proc. 22:93. doi: 10.1007/s11554-025-01668-0

[ref21] Gupta R. K. Jadeja D. Sahu Y. Kunhare N. (2026). Real-time lane detection for autonomous driving & GRAD-CAM visualization. Multim. Tools Appl. 85:116. doi: 10.1007/s11042-026-21291-w

[ref22] Hakani R. Rawat A. (2024). Edge computing-driven real-time drone detection using YOLOv9 and NVIDIA Jetson Nano. Drones 8:11. doi: 10.3390/drones8110680, 30654563

[ref23] Hashimoto K. Yokokawa Y. Arai N. Shen X. Zhang X. Raksincharoensak P. (2026). Signal temporal logic-based neural network for driving task generation in advanced driver assistance systems. Eng. Appl. Artif. Intell. 170:114164. doi: 10.1016/j.engappai.2026.114164, 38826717

[ref24] He Z. Wang K. Fang T. Su L. Chen R. Fei X. (2025). *Comprehensive Performance Evaluation of YOLOv11, YOLOv10, YOLOv9, YOLOv8 and YOLOv5 on Object Detection of Power Equipment.” 2025 37th Chinese Control and Decision Conference (CCDC)*, pp. 1281–1286.

[ref25] Hidayatullah P. Tubagus R. (2026). YOLO26: a comprehensive architecture overview and key improvements. arXiv 2026:14582. doi: 10.48550/arXiv.2602.14582

[ref26] Javeed M. A. Ghaffar M. A. Ashraf M. A. Zubair N. Metwally A. S. M. Tag-Eldin E. M. . (2023). Lane line detection and object scene segmentation using Otsu thresholding and the fast Hough transform for intelligent vehicles in complex road conditions. Electronics 12:1079. doi: 10.3390/electronics12051079

[ref27] Khanam R. Hussain M. (2024). YOLOv11: an overview of the key architectural enhancements. arXiv 2024:17725. doi: 10.48550/arXiv.2410.17725

[ref30] Liao Q. Chen J. Wang F. Rashid M. H. O. Xu T. Fan Y. (2025). Dual-path enhanced YOLO11 for lightweight instance segmentation with attention and efficient convolution. Electronics 14:3389. doi: 10.3390/electronics14173389

[ref31] Martins W. D. Osorio F. S. Bruno D. R. (2025). *Real Time Deep Learning Based Pothole Detection on Low Cost Embedded Devices for ADAS. 2025 Brazilian Conference on Robotics (CROS) 1 (April)*, pp. 1–6.

[ref32] Medina J. S. O. Lázaro J. G. M. Rassõlkin A. Ibrahim M. (2026). The road ahead: a comprehensive review of recent advances in traffic sign and lane line recognition for autonomous systems. IEEE Open J. Veh. Technol. 7, 160–178. doi: 10.1109/OJVT.2025.3635022

[ref33] Mohamed B. El Karch H. Jariri N. El gouri R. Hlou L. Mezouari A. (2023). *Strengthen Aircraft Real-Time Multiple Object Tracking with Optical Flow and Histogram of Oriented Gradient Provided HSMA Implemented in Low-Cost Energy VPU for UAV.” 2023 3rd International Conference on Innovative Research in Applied Science, Engineering and Technology (IRASET)*, pp. 1–5.

[ref36] Nandy M. Dharmesh Kirit D. (2026). Design and implementation of an embedded system for traffic sign detection and recognition system. AIP Conf. Proc. 3345:020101. doi: 10.1063/5.0298677

[ref37] Nguyen H. N. Luong T. N. Minh T. P. Hong N. M. T. Anh K. T. Hong Q. B. . (2025). Integration of multi-sensor fusion and decision-making architecture for autonomous vehicles in multi-object traffic conditions. Sensors 25:7083. doi: 10.3390/s25227083, 41305291 PMC12656283

[ref38] Ramos-Sanchez S. Lezama J. Yauri R. Zevallos J. (2026). Object detection on road: vehicle’s detection based on re-training models on NVIDIA-Jetson platform. J. Imaging 12:20. doi: 10.3390/jimaging12010020, 41590905 PMC12842717

[ref39] Raspberry Pi Hardware–Raspberry Pi Documentation. (2025). Available online at: https://www.raspberrypi.com/documentation/computers/raspberry-pi.html (Accessed September 22, 2025).

[ref41] Sanikommu V. Marripudi S. P. Yekkanti H. R. Divi R. Chandrakanth R. Mahindra P. (2025). Edge computing for detection of ship and ship port from remote sensing images using YOLO. Front. Artif. Intell. 8:8664. doi: 10.3389/frai.2025.1508664, 39981193 PMC11839658

[ref16] Santos Júnior E. S. Paixão T. Alvarez A. B. (2025). Comparative performance of YOLOv8, YOLOv9, YOLOv10, and YOLOv11 for layout analysis of historical documents images. Appl. Sci. 15:3164. doi: 10.3390/app15063164

[ref42] Sapkota R. Karkee M. (2026). YOLOE-26: integrating YOLO26 with YOLOE for real-time open-vocabulary instance segmentation. arXiv 2026:00168. doi: 10.48550/arXiv.2602.00168

[ref43] Sayyad J. Attarde K. (2026). A systematic literature review on deep learning approaches for small object detection. Array 29:100615. doi: 10.1016/j.array.2025.100615, 38826717

[ref44] Shaikh M. S. Faraz S. M. (2026). InSegNet: semantic segmentation-based lane detection network for clear and occluded roads. Ain Shams Eng. J. 17:103870. doi: 10.1016/j.asej.2025.103870

[ref46] Teboulbi S. Messaoud S. Hajjaji M. A. Atri M. Mtibaa A. (2026). Lightweight dual-YOLOv8 instance-aware semantic segmentation for real-time autonomous driving on edge ARM/GPU platforms. Int. J. Adv. Comput. Sci. Appl. 17:188. doi: 10.14569/IJACSA.2026.0170188

[ref47] Tian Y. Ye Q. Doermann D. (2025). YOLOv12: attention-centric real-time object detectors. arXiv 2025:12524. doi: 10.48550/arXiv.2502.12524

[ref48] Tsai S.-E. Yang S.-M. Hsieh C.-H. (2026). Real-time deterministic lane detection on CPU-only embedded systems via binary line segment filtering. Electronics 15:351. doi: 10.3390/electronics15020351

[ref49] Ultralytics. (2025a). *Segment*. Available online at: https://docs.ultralytics.com/fr/tasks/segment (Accessed March 18, 2025).

[ref50] Ultralytics. (2025b). *Ultralytics YOLO26*. Available online at: https://docs.ultralytics.com/fr/models/yolo26/ (Accessed December 6, 2025).

[ref51] Wang H. Wang J. Xiao B. Jiao Y. Guo J. *Drivable area and lane line detection model based on semantic segmentation*. 2024 IEEE 3rd International Conference on Computing and Machine Intelligence (ICMI), pp. 1–7. (2024).

[ref52] Zeng Z. Wang H. Yao C. Dong Z. Cai S. (2025). Optimizing surface defect detection with YOLOv9: the role of advanced backbone models. Front. Artif. Intell. 8:75154. doi: 10.3389/frai.2025.1675154, 41141912 PMC12549665

[ref53] Zhang G. Peng Y. Wang H. (2023). Road traffic sign detection method based on RTS R-CNN instance segmentation network. Sensors 23:6543. doi: 10.3390/s23146543, 37514837 PMC10385444

[ref54] Zhenbo B. Yang H. Fu Y. Zheng W. (2026). Enhanced YOLO-security deep learning model for ship detection in maritime security. Results Eng. 29:108815. doi: 10.1016/j.rineng.2025.108815

